# Familiar Disposition of May–Thurner Syndrome—A Case Series

**DOI:** 10.3390/life14020221

**Published:** 2024-02-04

**Authors:** Stefanie Nowak, André Jakob, Robert Dalla Pozza, Sebastian Michel, Nikolaus A. Haas, Joseph Pattathu

**Affiliations:** Department of Paediatric Cardiology and Paediatric Intensive Care, University Hospital, Ludwig-Maximilians-University, 81377 Munich, Germanynikolaus.haas@med.uni-muenchen.de (N.A.H.)

**Keywords:** May–Thurner syndrome, iliac compression syndrome, case series, hereditary, pulmonary embolism, childhood

## Abstract

May–Thurner syndrome is a venous compression syndrome of the pelvic vessels that represents a relevant risk factor for thrombus formation. The standard procedure to secure a diagnosis is venography, followed by endovascular therapy as the preferred treatment choice if the patient is symptomatic. In our case series, there are three related patients with May–Thurner syndrome. A 16-year-old female was admitted with pulmonary embolism, dyspnoea and hip pain. The compression syndrome was diagnosed with interventional venography, and the patient received venous stent implantation. Due to her family history, we also suspected her mother to be affected by the syndrome and elucidated the diagnosis shortly afterwards by invasive venography. Subsequently, we examined the patient’s 19-year-old brother, and magnetic resonance imaging confirmed May–Thurner syndrome. A similar case series has not been published before. In this case, the family relation indicates a possible hereditary aspect of May–Thurner syndrome. This hypothesis should be the subject of further research. In conclusion, it is essential to assess family history thoroughly when treating patients with May–Thurner syndrome.

## 1. Introduction

May–Thurner syndrome or iliac vein compression syndrome describes an anatomical variant leading to a symptomatic compression of the left common iliac vein between the right common iliac artery and the lumbar vertebrae [1]. In the following discussion, the mere anatomical appearance without clinical symptoms is called May–Thurner anatomy [1]. In 1957, an autopsy study by May and Thurner showed abnormal endothelial variations and alterations in the vein caused by those anatomical relations [2]. Based on Virchow’s findings concerning the formation of thrombi, such as change of blood flow, viscosity variation and endothelial irritation, known as Virchow’s Triad, May and Thurner recognised a possible cause for deep vein thrombosis occurring predominantly in the left lower extremity [2,3]. As of today’s literature, it is known that May–Thurner syndrome does not necessarily lead to thrombosis; the clinical presentation can vary from asymptomatic to chronicle venous insufficiency to pulmonary embolism in rare cases [2,4]. Recently, the number of publications on May–Thurner syndrome has increased due to greater awareness in clinical practice. Whereas there were often only individual case reports in the past, there are now more extensive studies and systematic reviews on the syndrome [5,6]. Another likely reason for the increase in syndrome diagnoses is the more accessible access to imaging diagnostics in daily clinical practice. However, the importance of this case series lies in our patients’ familiar relations. In the following, we present a female patient and her mother and brother, each diagnosed with May–Thurner syndrome.

## 2. Case Series

### 2.1. Case 1

First, we introduce a 16-year-old female patient admitted to our paediatric intensive care unit with bilateral pulmonary embolism and right ventricular strain. Previously, after a fall on her knee, the patient presented with dyspnoea and hip pain after several days of resting. As the patient’s mother suffered from a left-sided deep vein thrombosis at the age of 17, family history was suspected to be a positive risk factor, together with the patient’s use of oral contraceptives. The laboratory showed elevated D-dimer levels. After the confirmation of a pulmonary embolism by computed tomography, she was admitted to our intensive care unit under therapeutic anticoagulation with low molecular weight heparin and oxygen supplementation for further thrombolysis. The patient received alteplase (37.5 mg/h for 12 h) and, afterwards, intravenous heparin with an aimed PTT range of 40–60 s for treatment. On day 3, the patient was transferred to our intermediate care unit, and enoxaparin (2 × 80 mg) replaced the heparin treatment. Sonography of the femoral vein showed no signs of deep vein thrombosis or vascular compression. Since our patient reported, however, hip pain and dyspnoea before her sports accident, we suspected May–Thurner syndrome. This diagnosis was confirmed by magnetic resonance imaging, which showed compression of the left iliac vein by the right iliac artery and the fifth lumbar vertebrae. 

**Figure 1:**
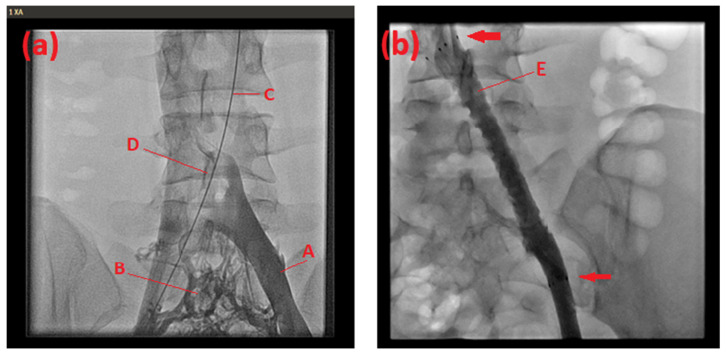
Venography of the 16-year-old patient (**a**) before and (**b**) after stent implantation. Left common iliac vein (A). Venous collaterals (B). Guide wire placed in the right iliac artery and aorta (C). Filling defect of contrast medium indicating the venous stenosis located at the arterial overcrossing (D). Left common iliac vein after balloon dilatation and stent implantation (E). Proximal and distal stent end (arrows).

A subsequent cardiac catheterization on day 6 showed pronounced stenosis and thrombosis of the left iliac vein, with a floating thrombus and a thrombus in the right pulmonary artery. Venous collaterals to the contralateral side were apparent (Figure 1a). Balloon dilatation (VACS II 16/40 mm) and lysis therapy (alteplase 75 mg/24 h), as well as heparin, were administered. Lysis was stopped after 10 hours because of headache and dizziness. Because of immediate re-stenosis observed in an invasive re-evaluation the following day, we performed a stent implantation (self-expandable, sinus Superflex 10/80 mm) in the left iliac vein (Figure 1b). A screening for thrombophilia showed no signs of Factor V Leiden or Prothrombin mutations. Hereafter, the patient received anticoagulation with vitamin K antagonists for one year, with an aimed INR value between two and three, and was discharged on day 10. A 4-month follow-up venography showed good patency in the vein; the patient merely reported mild discomfort during physical stress. Eventually, the anticoagulation was changed to rivaroxaban 20 mg. Two years after the stent implantation, stent patency was confirmed, and no further pulmonary embolism occurred. A whole exome sequencing was performed that showed no relevant clinical results. 

### 2.2. Case 2

Our second patient is the 49-year-old mother of Case 1. As mentioned before, she reported a left-sided deep vein thrombosis at the age of 17 when she was taking oral contraceptives. The 49-year-old woman suffered from tension in the left lower extremity and intermittent hip pain during long sitting periods. Those are typical symptoms of pelvic congestion syndrome. She wore compression stockings but did not take any anticoagulant medication. A thrombophilia screening was performed during pregnancy. The results was negative. Given her and her daughter’s medical history, and since May–Thurner syndrome is present in patients with pelvic congestion syndrome, we recommended screening for May–Thurner syndrome. Sonography of the iliac veins showed slow blood flow with higher turbulence in the left vein compared to the right side. 

**Figure 2:**
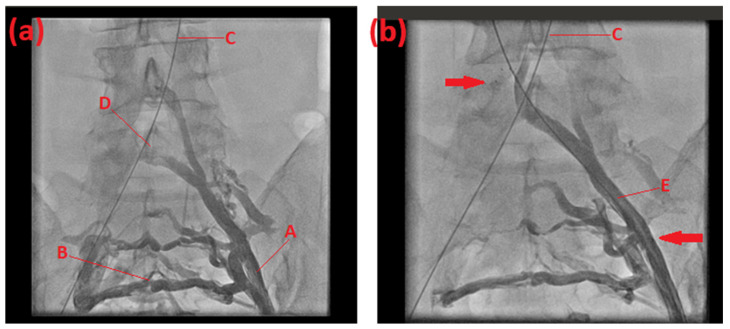
Venography of the 49-year-old mother (**a**) before and (**b**) after stent implantation. Left common iliac vein (A). Venous collaterals (B). Guide wire placed in the right iliac artery and aorta (C). Filling defect of contrast medium indicating the venous stenosis located at the arterial overcrossing (D). Left common iliac vein after balloon dilatation and stent implantation with placed guide wire (E). Proximal and distal stent end (arrows).

A loss of phasicity was observed on the left side. Therefore, the patient underwent invasive venography, where the diagnosis of May–Thurner syndrome was confirmed. The finding: subatretic stenosis with long segment narrowing of the left iliac vein due to the overcrossing right artery and subsequent thrombus formation was displayed, as well as severe venous collaterals to the contralateral side (Figure 2a). A pressure difference between the left- and right-sided femoral veins of 10 mmHg was measurable. We performed balloon dilatation (6/20 mm) and stent implantation (self-expandable, sinus Superflex 10/80 mm) in the left common iliac vein (Figure 2b). During and after the procedure, intravenous heparin treatment was administered as a prophylactic measure. The patient was discharged on day 5 and received anticoagulation with 100 mg of aspirin for 6 months. Additionally, we started vitamin K antagonists, according to our standard procedure for vein stenosis, with a target INR between two and three for 1 year. A 5-month catheter follow-up showed good patency with no evidence of in-stent-stenosis and no pressure gradient between the two legs, and the patient reported no more pain or discomfort.

### 2.3. Case 3

**Figure 3:**
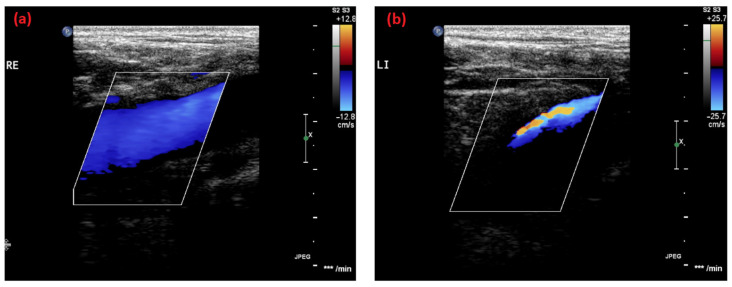
Ultrasound images of the (**a**) right and (**b**) left femoral vein of the 19-year-old brother. Turbulence and a decrease in vein diameter in the left vein compared to the right side indicate a venous compression syndrome.

The last patient is a 19-year-old man, the brother and son of our previous cases. He followed our recommendation regarding his mother’s and sister’s medical histories with thrombosis and was screened for May–Thurner syndrome. He reported no clinical symptoms regarding the lower extremities or possible thrombotic events. However, the sonography of the iliac veins showed compression of the left femoral vein, where a turbulent blood flow and a decrease in vein diameter compared to the right side were noticeable (Figure 3). The flow velocity was measured with approximately 20 cm/s in the right vein compared to 40 cm/s in the left. For further investigation, a magnetic resonance tomography revealed an apparent compression of the left proximal iliac vein between the right iliac artery and the fifth lumbar vertebrae. In addition, a pre-stenotic dilatation of the vein was detected. Therefore, the diagnosis of May–Thurner syndrome was confirmed. Since the patient was asymptomatic then, and anticoagulation therapy was not justified, we recommended antiplatelet prophylaxis with 100 mg of aspirin and a follow-up after one year. 

## 3. Discussion

May–Thurner syndrome was named after May and Thurner, whose studies in 1957 explored the microscopic alterations of the relevant part of the iliac vein for the first time. They discovered changes in the endothelial tissue of the venous lumen in areas of proximity to the overcrossing right iliac artery and described them as “spurs” [2]. Their findings amplified the 1908 macroscopic research conducted by McMurrich, who spoke about “adhesions” in the iliac vein and made assumptions about a possible correlation to left lower extremity thrombosis [7]. The first clinical studies were performed in the 1960s, and the name iliac vein compression syndrome was established [8]. Nowadays, both terms are used synonymously. 

The majority of patients with May–Thurner syndrome remain asymptomatic. Kibbe et al. examined the existence of iliac vein compression in an asymptomatic patient cohort. Their results indicate that 90% of those patients have subclinical compression of their veins. They also describe women as having a higher risk for more severe compression [9]. Meanwhile, the presentation of symptomatic patients includes leg pain and oedema, deep vein thrombosis of the lower extremity, post-thrombotic syndrome, and, in rare cases, also dyspnoea due to pulmonary embolism. Children especially show many nonspecific symptoms, e.g., abdominal pain [10,11,12,13]. As seen in our case report, the first clinical appearance might also be in the context of severe pulmonary embolism, which is not a common complication [4]. The symptoms reported by our 49-year-old patient go along with pelvic congestion syndrome. It consists of chronic pain in the pelvis due to venous insufficiency and structural variants. 

Amongst other vascular syndromes, May–Thurner syndrome was described as being associated with pelvic congestion syndrome [14,15]. It is, therefore, essential to identify the underlying venous compression syndrome to gain therapeutic success. The 49-year-old mother is in line with the often reported combination of age and gender, being female and in her forties [6]. Gender differences are often the subject of research. However, there are few comparisons between racial and ethnic groups. There are several studies from Europe, America and Asia [6,16]. It would be interesting to compare the syndrome’s incidence in different racial groups. The children, both adolescents, match the patient characteristics often-reported in paediatrics [10]. 

The external compression and the endoluminal changes described are risk factors for venous thrombosis. However, it is essential to emphasise that a combination of May–Thurner syndrome with other thrombo-embolic risk factors occurs frequently. A recent meta-analysis of paediatric data concerning May–Thurner syndrome shows that 66% of the female patients took oral contraceptives, similar to our 16-year-old female patient. Thrombophilia is also an additional risk factor in several cases [10]. Thrombophilia testing in our first two patients showed no results. We performed whole exome sequencing in Case 1, but it showed no relevant clinical results, and whether there was a correlation with May–Thurner syndrome remained questionable. This could be a target for further research. 

Furthermore, the effects of iliac compression syndrome go beyond mere coagulation disorders. Al-Otaibi et al. reported a very high incidence of May–Thurner anatomy (29.7%) in patients with chronic thromboembolic pulmonary hypertension referred for surgical pulmonary endarterectomy [17]. This may confirm the importance of regular screening for this anatomical variation and subsequent treatment in patients with pulmonary embolisms. 

The most important measures for the diagnostic workup of iliac vein compression syndrome are imaging procedures. Ultrasound may not always ensure the diagnosis; however, it is a fast and non-invasive method to uncover the first information about a possible May–Thurner syndrome diagnosis. Differences in venous flow in a side-by-side comparison may indicate venous compression [18]. The mother and her son observed differences in the venous blood flow and diameter discrepancy between the left and right femoral veins. The alternatives are magnetic resonance imaging of the pelvic vessels or intravenous contrast venography. The latter permits the confirmation of the diagnosis and an immediate therapeutic intervention [19]. Therapeutic success depends on sufficient patency of the veins, together with a risk reduction of restenosis or recurring thrombosis. The therapies in paediatric patients with May–Thurner syndrome follow the same approach as in adult patients. The standard treatments include local or systemic lysis, balloon dilatation of the venous stenosis and venous stent implantation [10,13,20]. Oguzkurt et al. observed a successful endovascular therapy in 34 out of 36 patients. Their primary patency rate after four years was 80% [21]. Ten patients received stent implantation in a paediatric cohort of the Mayo Clinic, Minnesota, where patency at follow-up (1.1 years) was achieved in 9 out of 10 children; these include 5 patients with secondary stent recanalisation [13]. Given the age of the first patient and the consequences associated with stent implantation, balloon dilatation was our first choice for treatment. However, after restenosis, we decided to proceed with stenting. For the mother, stenting was the first choice. In both cases, the procedure was successful without complications and provided sufficient recanalisation and perfusion. The therapeutic success of our 49-year-old patient, where treatment reduced our patient’s pain significantly, even several years after her thrombotic events, encourages the diagnosis and treatment of May–Thurner syndrome. 

Considering this case series, a familiar accumulation of May–Thurner syndrome is likely. However, current research gives hardly any information on the subject. Familial clustering is not described in the literature on May–Thurner syndrome. A single case report in 1973 discusses two sisters, each presenting with deep vein thrombosis in the lower limb and subsequently diagnosed with iliac vein compression syndrome [22]. Our case series indicates that May–Thurner syndrome occurs in a hereditary pattern. This aspect requires further exploration in future studies. 

## 4. Conclusions

This case series indicates the possibility of a hereditary aetiology of May–Thurner syndrome. It is essential to take a detailed family history when children, adolescents or young adults present with (left-sided) iliac thrombosis and/or pulmonary embolism. We recommend enquiring about thrombotic and embolic events in the immediate family and whether a cause could be found, as May–Thurner syndrome should be considered, particularly in the case of unexplained thromboses. Confirming the diagnosis of an iliac vein compression syndrome and subsequent management could increase the long-term prognosis significantly, since modern treatments safely allow the relief of the obstruction, symptom control and/or prophylaxis in the event of additional transient risk factors. However, caution is necessary to prevent diagnostics and overtreatment in asymptomatic cases. Future research is required to address the hereditary aspects of this syndrome in order to validate this hypothesis.

## Data Availability

Data can be shared by personal request to the corresponding author.
